# A Case of Rupture of the Bladder, Terminating Fatally on the 4th Day

**Published:** 1830

**Authors:** John E. Bush

**Affiliations:** Cincinnati


**Art.—VI.** *A case of rupture of the bladder terminating fatally on the 4th day.* By John E. Bush, M. D., of Cincinnati.

I was sent for, on the 21st June to visit Mr. H. who was represented to have injured himself very seriously by a fall a short time before. As I was absent when the messenger arrived, my pupil, Mr. Andrews, called to see the patient. He found him lying on his face upon the floor, in very great pain, and absolutely refusing to be moved; and was informed that he was intemperate in his habits; and that he had returned home on the evening before somewhat intoxicated. On getting up in the morning, about 10 o'clock, he had fallen across the bed post, and complained immediately of most excruciating pain about the abdomen. As he positively refused to submit to any remedial measures, Mr. A. left him; and having been again sent for, I saw him on the next morning. I found him suffering great pain throughout the whole abdominal region, his pulse quick, frequent, tense and rather strong, the abdomen swelled and tense—a constant inclination to void his urine without the power of doing it—with a sensation which he expressed by saying "he felt as if his bladder would burst". His bowels had not been moved since he received the injury.

By introducing the catheter about half a pint of urine was drawn off; the loss of thirty oz. of blood produced a disposition to syncope and a complete reduction of the pulse. Twenty grains calomel were given to be followed by castor oil and spts. of turpentine each half oz. every hour, until evacuations were produced. Fomentations to the abdomen.

Evening. No relief from the pain; no alvine evacuations; pulse increased in frequency, but soft and weak; skin of the natural temperature; a small quantity of urine was discharged by the catheter; bleeding was attempted but the pulse gave way under the loss of six oz. Fomentations continued during the night.

23d. Tension and soreness of the abdomen increased. Spasms of the abdominal muscles with excruciating pain, shooting from the scrobiculus cordis to the loins, produced by the slightest motion. Tongue slightly furred. Catheter again introduced—a blister applied to the abdomen, and infusion of senna with epsom salts directed during the day, their operation to be assisted by enemata.

Evening, slight alvine evacuations healthy in their appearance; reported to have had a free voluntary discharge of urine. No alleviation of the symptoms, pulse 150, not contracted, but without tension or strength.

Hitherto we had been led to suspect a visceral læsion, and our suspicions were early directed to the bladder, but the voluntary discharge of urine now led us to think differently. At the recommendation of Dr. Finley, who met me in consultation, he was again bled. The loss of eight oz. reduced the pulse and relaxed the system very much. The pain was somewhat alleviated, and after a large dose of denarcotised laudanum, he passed the night in comparative comfort. In addition to the laudanum were given 20 grs. calomel.

24th. Pain somewhat relieved when perfectly at rest. Calomel and opium directed to be taken during the day and the bowels evacuated by enemata.

Evening, much worse ; pulse nearly imperceptible, extremities cold, no pain except on pressure, slightly delirious; during the night these unfavorable symptoms increased, and he died about 5 o'clock on the next morning. To the last he retained a considerable degree of muscular strength, and within 5 minutes of his death, he arose from his bed, walked across the room and poured out a tumbler of water.

*Appearances on dissection.* On opening the abdomen a large quantity of transparent fluid presented itself (perhaps a gallon and a half) very offensive, and supposed by some present to have a urinous smell ; the bladder was ruptured at the fundus, and closely contracted on the pubes ; the intestines covered with coagulable lymph, agglutinated to each other and their peritoneal coat in a state of high inflammation ;—the mucous surface not altered from the usual appearance ; the omentum dark colored, but not gangrenous.

The reader will no doubt be struck with the fact, that the constitutional symptoms in this case were rather those of irritation, than inflammation. Although the local symptoms urgently called for depletion, the pulse and the state of the surface, neither manifested the usual symptoms of inflammatory action, not of that depression of the vital powers, which accompanies violent visceral inflammation ; bearing in this respect a close analogy to the cases of rupture of the duodenum, and of the pylorus, reported by Dr. Drake in former numbers of this journal.

The facility, with which the bladder was ruptured is also worthy of notice, and can be accounted for, only by the supposition of great distension, and of that passive state of its contracting fibres which follows as the consequence of a fit of intoxication.

